# Genetic determinants of antidepressant and antipsychotic drug response

**DOI:** 10.1007/s00406-024-01918-5

**Published:** 2024-10-09

**Authors:** Hans H. Stassen, S. Bachmann, R. Bridler, K. Cattapan, A. M. Hartmann, D. Rujescu, E. Seifritz, M. Weisbrod, Chr. Scharfetter

**Affiliations:** 1 Institute for Response-Genetics, Department of Psychiatry, Psychotherapy and Psychosomatics, Psychiatric University Hospital, Zurich, CH-8032 Switzerland; 2 Department of Psychiatry, Geneva University Hospitals, Thônex, CH-1226 Switzerland; 3 Department of Psychiatry, Psychotherapy, and Psychosomatics, University of Halle, Halle, D-06112 Germany; 4 Clienia AG, Psychiatric Hospital, Littenheid, CH-9573 Switzerland; 5 Sanatorium Kilchberg, Alte Landstrasse 70, Kilchberg, CH-8802 Switzerland; 6 University Hospital of Psychiatry and Psychotherapy, University of Bern, Bern, CH-3012 Switzerland; 7 Clinical Division of General Psychiatry, Medical University of Vienna, Wien, A-1090 Austria; 8 Department of Psychiatry, Psychotherapy and Psychosomatics, Psychiatric University Hospital, Zurich, CH-8032 Switzerland; 9 Department of General Psychiatry, Center of Psychosocial Medicine, University of Heidelberg, Heidelberg, D-69115 Germany; 10 SRH Hospital Karlsbad-Langensteinbach, Karlsbad, D-76307 Germany

**Keywords:** Antidepressants, Antipsychotics, Depression, Schizophrenia, Response to treatment, Efficacy, Side effect profiles, Gene vectors, Genotypic patterns, Genetic diversity, Molecular-genetic neural nets

## Abstract

Today, more than 90% of inpatients hospitalized with Major Depression or Schizophrenia are treated with psychotropic drugs. Since none of the treatment options is causal, response rates are modest and the course of recovery is very heterogeneous. Genetic studies on the etiology and pathogenesis of major psychiatric disorders over the past decades have been largely unsuccessful. Likewise, genetic studies to predict response to psychopharmacological treatment have also not been particularly successful. In this project we have recruited 902 inpatients with ICD-10 diagnoses of schizophrenic (“F2 patients”) or depressive disorders (“F3 patients”). The study assessed today’s acute inpatient treatment regimens with up to 8 repeated measurements regarding the time course of recovery and adverse side effects. The genotyping included 100 candidate genes with genotypic patterns computed from 549 Single Nucleotide Polymorphisms (SNPs). To predict response to psychopharmacological treatment, we relied on a multidimensional approach to analyzing genetic diversity in combination with multilayer Neural Nets (NNs). Central to this new method were the “gene vectors” that (1) assessed the multidimensional genotypic patterns observed with genes; and (2) evaluated the correlations between genes. By means of these methods, we searched for combinations of multidimensional genotypic patterns that were characteristic of treatment responders while being rare among non-responders. The chosen method of approach provided a powerful technique to detail the complex structures of SNP data that are not detectable by conventional association methods. Molecular-genetic NNs enabled correct classification of 100% “non-responders”, along with 94.7% correctly classified “responders” among the F2 patients, and 82.6% correctly classified “responders” among the F3 patients. The F2 and F3 classifiers were not disjoint but showed an overlap of 29.6% and 35.7% between the diagnostic groups, thus indicating that clinical diagnoses may not constitute etiologic entities. Our results suggested that patients may have an unspecific physical-genetic disposition that enables, facilitates, impedes or prevents recovery from major psychiatric disorders by setting various thresholds for exogenous triggers that initiate improvement (“recovery disposition”). Even though this disposition is not causally linked to recovery, it can nonetheless be clinically used in the sense of a “surrogate”. Indeed, clinicians are also interested in reliable tools that can “do the job”, despite the fact that etiology and pathogenesis of the treated disorders remain unknown.

**Supplementary Information:**

The online version contains supplementary material available at 10.1007/s00406-024-01918-5.

## Introduction

Today, more than 90% of inpatients hospitalized with Major Depression or Schizophrenia are treated with psychotropic drugs. The majority of them receive combinations of several antidepressants, antipsychotics, mood stabilizers, anxiolytics, hypnotics, antihistamines, and anticholinergics, along with other somatic treatments [1]. Since none of the treatment options is causal, response rates are modest (in the range of 35-45%), and the course of recovery is very heterogeneous. About one third of patients get “stuck” after initial improvement, while some 20% do not show any improvement at all. And, in the case of schizophrenia, antipsychotics reduce the positive symptoms very quickly, whereas general psychopathology and negative symptoms are only partially alleviated [2].

In tandem with the drug treatments comes the burden of adverse reactions in terms of cardiovascular and neurological disturbances, along with impairments of sleep and excessive weight gain [3–10]. Between 50% and 70% of patients experience such troublesome and highly undesirable side effects. From a clinical point of view, it is most frustrating that no reliable prediction can be made as to if and when a particular patient will respond to a particular treatment, or if and what side effects he/she will develop under the chosen treatment.

Genetic studies on the etiology and pathogenesis of major psychiatric disorders over the past decades have not yet led to the hoped-for breakthrough. There are 100 + psychiatry-relevant Genome-Wide Association Studies (“GWASs”) whose results have been compared by several review articles [11]. Reproducibility appears to be a central problem as every study finds something different, whether for clinical diagnoses such as depression [12], schizophrenia [13], bipolar disorder [14], and Alzheimer’s disease [15], or for endophenotypes instead of clinical diagnoses [16]. Even GWASs that used the same data material, but were carried out by different research groups, exhibited inconsistencies in the outcome [15] (for details see Appendix). Particularly disillusioning is the fact that GWAS results produced with gigantic effort explain far less than 10% of the observed phenotypic variance.

The reproducibility of GWASs can get compromised because (1) it is difficult to correctly interpret associations: signals with strong association values may be “false-positives” while signals with weak association values may be “false-negatives”; (2) the phenotypic variance explained by individual SNPs is tiny and non-additive, so that sufficiently-powered GWASs require thousands of cases and controls [17]; (3) the “nearest gene” concept of GWASs complicates the cross-comparison of results excessively [18]; (4) interrelations between genomic loci are not taken into account and multidimensional genotypic patterns are not searched for [19]; and (5) clinical diagnoses used as phenotype do not represent etiological entities [17].

Genetic studies to predict response under psychopharmacological treatment [20–26] or to predict excessive weight gain induced by psychopharmacological treatment [27–30] have also not been particularly successful to date. This despite the high expectations raised by GWASs more than a decade ago: “GWAS of antidepressant treatment outcome may ultimately help match medications with patients, maximizing efficacy while minimizing adverse effects” [31]. Similarly, the many studies with candidate genes or candidate SNPs (Single Nucleotide Polymorphisms) that have been carried out to date explained only small proportions of the phenotypic variance observed with psychotropic drug response, while the respective SNPs have rarely ever been reproducible [32–37].

All in all, it does not look as if the question of etiology and pathogenesis in connection with major psychiatric disorders could be answered in the near future, so that no causal treatments can be expected either. In consequence, reliable prediction of non-response to psychotropic drugs would avoid needless treatments and unnecessary side effects for patients.

Given this situation, it seemed opportune to consider alternatives to the standard approaches to modeling psychotropic drug effects, as well as to question the model of a direct causal influence of genes on psychopharmacological treatment response, as such models are difficult to reconcile with clinical data and essentially fail to “explain” the observed between-subject variance. By contrast, clinical studies with thousands of patients suggested, that instead of inducing causal effects, active substances merely initiate improvement in the sense of “trigger effects”. Once “triggered”, the patients’ further course of improvement followed its “natural” pattern, irrespective of the active substances’ biochemical design and primary site of pharmacological action [38–41]. These findings strongly supported the view that patients possess an unspecific physical-genetic disposition that enables, facilitates, impedes or prevents recovery from major psychiatric disorders by setting various thresholds for exogenous triggers that can initiate improvement. This «recovery disposition»^1^ is presumably not causally linked to patients’ response to therapeutic interventions, but can nonetheless be used in the sense of a “surrogate”, since clinicians are also highly interested in reliable tools that can “do the job”, even though they do not allow causal conclusions to be drawn.

Unlike standard association methods, this study therefore addressed the question of the extent to which irregularities in genetic diversity (1) are involved in the «recovery disposition»; and (2) might be related to the patients’ response to therapeutic interventions.

Genetic diversity can be assessed through multidimensional “gene vectors” so that systematic analyses of the patients’ genotypic patterns, made up by the “gene vectors”, will reveal any irregularities in genetic diversity. The “gene vectors” are assembled from 4 to 8 polymorphic SNPs located within genes and representing the genes’ distinctive “fingerprints”. As SNPs can exhibit three different expressions (regardless of allele definition), each SNP can take on one of three different binary patterns: 0/0, 0/1, or 1/1. With *m* SNPs, a total of 3^*m*^ different genotypic patterns is theoretically observable per gene vector: 81 patterns for 4 SNPs, 243 patterns for 5 SNPs, …. 6,561 patterns for 8 SNPs. The minimum sample size required to actually observe that number of different genotypic patterns can be estimated. Under the assumption of equally distributed SNPs, a sample size of 4 times 3^*m*^ is expected to exhibit about 95% of the theoretically possible patterns.

Specifically, on the basis of data of 902 inpatients treated for major depression or schizophrenia, this project analyzed irregularities in genetic diversity by means of multidimensional genotypic patterns and multilayer neural nets (“NNs”) in order to separate (1) treatment “responders” from “non-responders”; (2) “early improvers” from “late improvers”; and (3) patients with normal levels of Immunoglobulin M (IgM) from patients with chronically elevated IgM levels (indicating latent inflammation [42]).

## Materials

In a “naturalistic” observational study of psychiatric inpatients, we recruited 902 patients hospitalized at five residential mental health treatment centers with an ICD-10 diagnosis of either schizophrenic (*n* = 264; “F2 patients”) or depressive disorders (*n* = 638; “F3 patients”). New admissions with a suspected primary ICD-10 diagnosis of “F2x.x” or “F32.x/F33.x” were invited to enroll in the study. All participating patients signed a written “informed consent” after having been informed about the aims of the project, and that they can stop their participation at any time without any disadvantages. Final diagnoses were decided by two senior psychiatrists.

The study protocol included (1) up to 8 repeated measurements over 6 weeks assessing the time course of improvement through the 17/21-item Hamilton Depression Scale HAM-D [43] or the 30-item Positive and Negative Syndrome Scale PANSS [44]; (2) up to 8 repeated measurements over 5 weeks assessing medication and unwanted side effects through the 46-item Medication and Side Effects Inventory MEDIS [45]; and (3) the collection of blood samples for serum extraction and DNA isolation. Psychopathology was assessed by specifically trained psychiatrists and psychologists to improve inter-rater agreement.

A minimum baseline score of at least 15 on the HAM-D17 Scale (primary “F32.x/F33.x” diagnoses), or of at least 21 on the general psychopathology PANSS-G Scale (primary “F2x.x” diagnoses), was required at entry into study. The PANSS-G scale was chosen in order to prioritize illness-related disabilities in daily functioning over acute productive symptomatology and longer persisting negative symptoms. Baseline severity was categorized in the following way; among F3 patients: HAM-D17 < 20 as “mild”; HAM-D17 from 20 to 24 as “moderate”; and HAM-D17 > 24 as “severe”; among F2 patients: PANSS-G < 30 as “mild”; PANSS-G from 30 to 40 as “moderate”; and PANSS-G > 40 as “severe”. In line with our previous studies in this field [39, 40] we used scale-based cutoff values for the definition of response to treatment. “Response” under schizophrenia therapies was defined by a sustained 40% PANSS-P baseline score reduction and under depression therapies by a sustained 50% HAM-D17 baseline score reduction^2^. Similarly, we defined “onset of improvement” by a sustained 20% PANSS-P, or a 20% HAM-D17 baseline score reduction, respectively. Patients who showed onset of improvement within the first 2 weeks of treatment were referred to as “early improvers”.

### Ethics

The study was approved by the local ethics committees in charge of the participating centers. Written informed consent was obtained from all participants.

### Genotyping

Critically important for the envisaged analyses of genetic diversity is the selection of informative genes whose “gene vectors” and genotypic patterns derived from them (1) possess sufficient variation across subjects so that subtle between-subject differences can be resolved; and (2) provide access to the postulated irregularities in genetic diversity. Due to lack of suitable a priori knowledge, the gene selection for this study was necessarily a kind of “blind shot in the dark” and mostly included genes reported in the literature as “possibly” involved in the pathogenesis of psychiatric disorders (without the existence of reliable probability estimates). Luckily enough, the selection of the 100 candidate genes in preparation for this study included quite a number of highly informative genes, so that the study could be completed successfully. The genes’ genotypic patterns were assessed through 549 Single Nucleotide Polymorphism (SNPs).

Genotyping was performed using the iPLEX assay on the MassARRAY MALDI-TOF mass spectrometer “Sequenom” [46], multiplexed with 40 + separate loci per reaction. This method is based on single base extension (SBE) of SNP specific primers using mass modified ddNTPs. In addition, SBE primer length was used to ensure unambiguous resolution of SNPs and alleles. Quality criteria were a sample call rate > 80%, SNP call rate > 95%, and genotypes of CEU Trios in accordance with HapMap database > 98%.

Cytokine data (*Il-6*, *TNF-a*, *Neopterin*, *TGF-β1*, and *sCD14*) as well as IgM levels were determined from the serum (IgM by Viollier Laboratories Basel).

### Quantifying genetic diversity

The quantification of genetic diversity inherent in a population relied on “gene vectors” which were assembled per gene from the genotypes of 4–8 polymorphic SNPs located within each gene. As a SNP can exhibit three different expressions regardless of allele definition, a base-3 system was used to construct gene vectors:

$$\eqalign{& {\rm{gene}}\,{\rm{vector:}}\,v_i^{{\rm{(}}j{\rm{)}}} = \sum\limits_{k = 1}^{m{\rm{(}}j{\rm{)}}} {s_{ik}^{(j)}} {3^{k - 1}} \cr & i = 1,2,\, \ldots \,{\rm{N}} \to {\rm{subjects}} \cr & j = 1,2,\, \ldots \,{\rm{M}} \to {\rm{genes}} \cr & {\rm{s}}_{ik}^{{\rm{(}}j{\rm{)}}} \in {\rm{\{ }}0,1,2{\rm{\} }} \to {\rm{SNPs}} \cr & m{\rm{(}}j{\rm{)}} \to {\rm{number}}\,{\rm{of}}\,{\rm{SNPs}}\,{\rm{in}}\,{\rm{the}}\,j{\rm{ - th}}\,{\rm{gene}} \cr}$$

With *m* SNPs, a total of *3*^*m*^ different genotypic patterns would be theoretically possible per gene. Under the assumption of equally distributed SNPs, a sample size of 4 times *3*^*m*^ is expected to exhibit about 95% of the theoretically possible patterns. This estimate was almost perfectly confirmed by simulations based on the study’s genes and SNP selections. The number of different genotypic patterns observed with “gene vectors”, the genes’ so-called “diversity index”, served as estimate of a population’s inherent genetic diversity.

### Statistical analyses

We used the Statistical Analysis Software SAS/STAT 9.4 by SAS Institute Inc. for repeated measurement analyses (PROCs ANOVA, CORR(PEARSON/SPEARMAN), FREQ(CHISQ), GLM, NPAR1WAY, TTEST), and the SPSS 28 Statistics Package by IBM, along with PROC *HPNEURAL* from SAS Enterprise Miner 15.1, for Neural Nets analyses.

### Neural nets

**Fig. 1:**
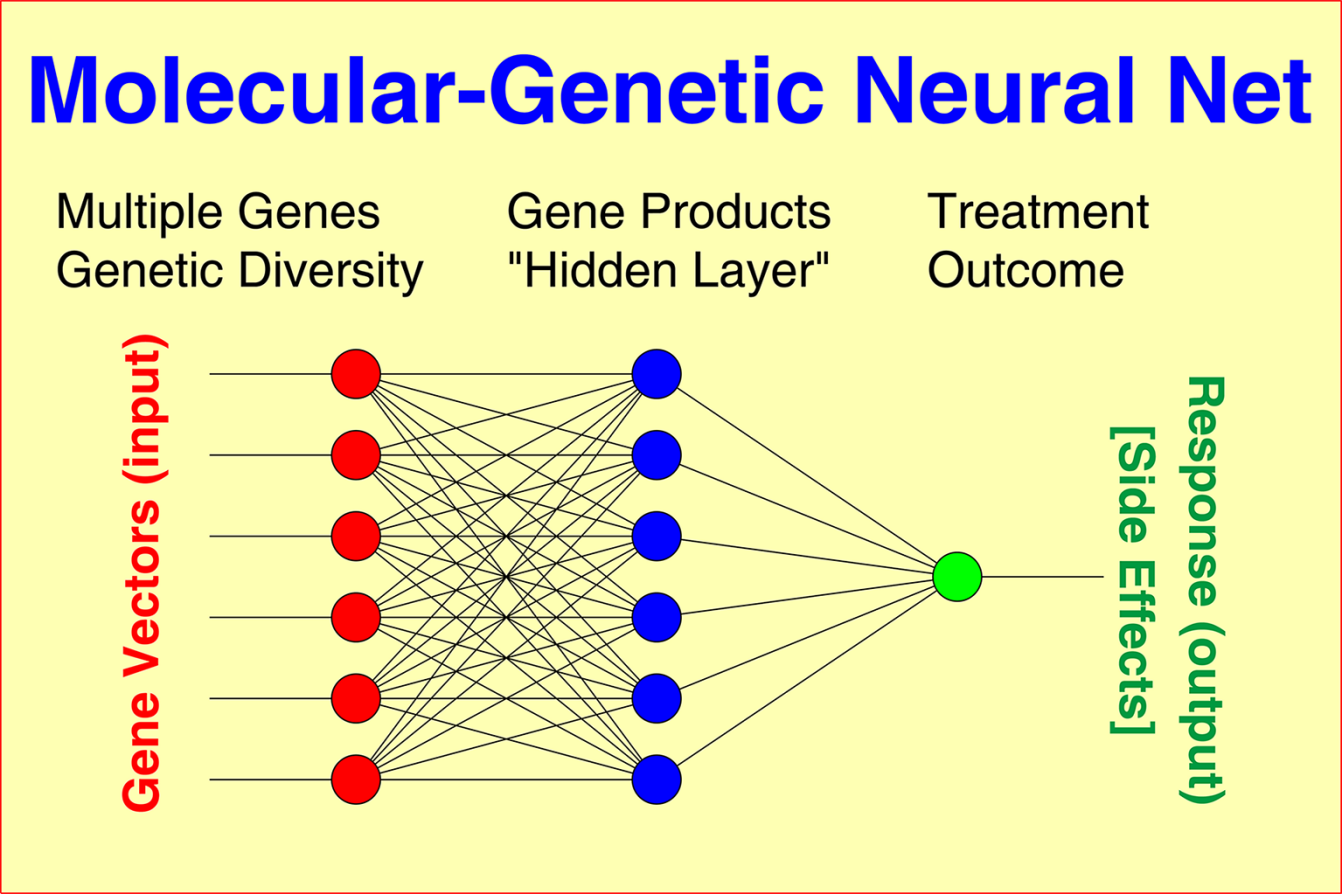
Principal schema of a multilayer Neural Net (NN) where response to treatment and unwanted side effects (output) is predicted from multiple gene vectors (input) connected to each other by complex interactions via one or more “hidden” layer(s). The NN algorithm iteratively constructs a model that is simultaneously fitted to the observed data of all patients. The achievable goodness of fit depends on the information included, the quality of underlying data, and the number of intermediate layers implemented to model nonlinear interactions

Nonlinear Neural Nets (NN) connect the “neurons” of input and output layers via one or more “hidden” layers (Fig. 1), thus featuring a relatively large number of free parameters.

NN connections are realized through (1) weight matrices and (2) model fitting algorithms minimizing an error function in the weight space (goodness of fit). All outputs are computed using sigmoid thresholding of the scalar product of the corresponding weight and input vectors. Outputs at stage “*s*” are connected to each input of stage “*s* + 1”. The most popular model fitting strategy, the backpropagation algorithm, looks for the minimum of the error function using the method of gradient descent (“steepest descent”). The basic algorithm is [47]:

$$\eqalign{& {\rm{(i)}}\,{\bf{Output:}}\,{s_i} = \sigma \left[ {\sum\limits_j {{w_{ij}}{s_j}} } \right] \cr & {s_i}{\rm{:}}{y_i}\,{\rm{observed}}\,\,{\rm{(}}i{\rm{ = 1,2,}} \ldots {N_i}{\rm{)}} \cr}$$

$$\eqalign{& {\rm{(j)}}\,{\bf{Hidden}}\,{\bf{layer}}{\rm{:}}\,{s_j} = \sigma \left[ {\sum\limits_k {{w_{jk}}{s_k}} } \right] \cr & {\rm{(}}j{\rm{ = 1,2,}} \ldots {N_j}{\rm{)}} \cr}$$

$${\rm{(k)}}\,{\bf{Input}}{\rm{:}}\,{s_k} = {x_k}\,\,{{\rm{x}}_k}\,{\rm{observed}}\,\,{\rm{(}}k{\rm{ = 1,2,}} \ldots {N_k}{\rm{)}}$$

$$\eqalign{& {\bf{Improvements:}}\,\Delta {w_{ij}} = \alpha \cdot \varepsilon _i^v \cdot {s_j} \cdot {s_i}{\rm{(1 - }}{s_i}{\rm{)}} \cr & \varepsilon _i^v = y_i^v - s_i^v\,\,\,{\rm{(v = 1,2,}} \ldots p{\rm{)}} \cr}$$

$$\Delta {w_{jk}} = \alpha \cdot \sum\limits_{i = 1}^{{N_I}} {\varepsilon _i^v \cdot {s_k} \cdot {s_i}{\rm{(1 - }}{s_i}{\rm{)}} \cdot {{\rm{w}}_{ij}} \cdot s_j{\rm{(1 - }}{s_j}{\rm{)}}}$$

where *x*_k_ denote observed stimuli, *y*_j_ observed responses, σ the activation function of sigmoid-type: R→(0,1), *α* the learning rate, and *p* the number of probes (patients). The achievable precision of the model depends on the information included, the quality of underlying data, and the number of intermediate layers implemented to model nonlinear interactions.

Results derived through standard NN approaches, which use 80% of samples for training and the remaining 20% for testing tend to be over-optimistic, in particular in the presence of assessment errors and missing data. By contrast, the *k*-fold cross-validation approach splits the data into *k* roughly equal parts, using *k*-1 partitions for training, while one partition is used for testing. This process is repeated until each partition has served as a testing set, so that *k* estimates of prediction errors are generated. The resulting prediction errors are approximately unbiased for the “true” error for sufficiently large *k* (*k* ≈ 10 is a typical value in practice). In consequence, we relied on the *k*-fold cross-validation strategy with *k* = 10 throughout the entire project and applied the well-proven “random walk” strategy in order to distinguish between local and global minima.

### A priori knowledge derived by machine learning techniques

This study did not use machine learning techniques to derive classifiers “ex nihilo” but, rather, followed a two-stage approach: (1) using standard Artificial Intelligence (AI) procedures, we screened all candidate genes for multidimensional genotypic patterns that were characteristic of treatment responders while being rare among non-responders (< 10%), and vice versa; (2) only genes that met these criteria (“response genes”) entered into subsequent Neural Net analyses (NNs).

## Results: genetic diversity

The evaluation of the gene vectors based on 549 genotyped SNPs resulted in a total of 7,748 different genotypic patterns, which was only one third (33.8%) of the theoretical number expected for this sample size under the assumption of uncorrelated SNPs. This indicated the existence of significant between-SNP correlations.

In addition to the between-SNP correlations, we also found between-gene correlations. In fact, genotypic patterns observed with a pair of genes must not necessarily be independent of each other but may be “linked” to some extent, so that genotypic patterns can co-occur more often than expected by chance, even across chromosomes. Almost one third of genes showed such correlations, with mean correlations around 0.107 ± 0.102 across diagnostic subgroups.

The number of different genotypic patterns per gene (“diversity index” *d*) was found to be *d* = 80.5 on average (range 7-341), while being only weakly correlated with the constituent number of SNPs. A generalized linear regression model GLM of constituent SNPs explained no more than about 20% of the observed variance, whereas the combined factors chromosome, gene size, and gene position explained some 6%. All this suggested that genetic diversity most likely reflects an intrinsic gene property that has emerged over the course of evolution. It is worth noting that the diversity indices provide a direct measure of the genes’ information content regarding the resolution of between-subject differences: a diversity index of *d* = 7 indicates almost no between-subject variation (most subjects have identical genotypic patterns), whereas a diversity index of *d* = 341 means a high between-subject variation since there were 341 different genotypic patterns observed in the sample under investigation.

About two thirds of the genes exhibited 8–12 prominent genotypic patterns that showed up in more than 80% of study patients, in a similar way across various subgroups including “*responders”*, “*non-responders”*, “*early improvers”*, “*mild cases”*, “*severe cases”*, “*no side effects”*, “*severe side effects”*, “*normal IgM level”*, and “*chronically elevated IgM level”* among others (Table 1). The CLOCK gene may serve as an example (Table 2).

**Table 1:** About two thirds of the 100 candidate genes of this study exhibited 8–12 prominent genotypic patterns (left row) that showed up in more than 70% of study patients (bold lines), in a similar way across various subgroups p1-p20, including “responders”, “non-responders”, “early improvers”, “mild cases”, “severe cases”, “no side effects”, “severe side effects”, “normal IgM level”, and “chronically elevated IgM level” among others. The CLOCK gene is given here as an example

CLOCK Gene: Distribution of Genotypic Patterns in 20 Populations p1-p20																				
Pattern	p1	p2	p3	p4	p5	p6	p7	p8	p9	p10	p11	p12	p13	p14	p15	p16	p17	p18	p19	p20
0	4	0	0	0	4	2	4	2	0	2	0	0	2	1	1	0	0	4	2	2
15,925,296	3	0	0	0	1	2	2	0	0	0	0	0	0	0	0	0	0	0	3	0
15,925,308	4	0	1	1	1	2	3	0	0	0	0	0	0	3	1	0	1	0	3	0
16,711,728	5	2	1	2	1	1	1	0	0	0	0	0	0	2	0	0	3	1	1	0
**16,711,740**	**86**	**23**	**6**	**13**	**39**	**15**	**26**	**6**	**20**	**8**	**18**	**6**	**20**	**24**	**17**	**3**	**27**	**17**	**39**	**17**
16,711,742	2	0	0	0	2	0	0	0	0	0	0	0	0	0	1	0	0	1	1	0
149,618,856	7	3	2	4	1	1	1	0	0	0	0	0	0	1	2	0	5	0	2	0
178,956,986	3	2	0	0	0	1	1	0	0	0	0	0	0	1	1	0	2	0	1	0
214,106,304	2	0	0	0	1	1	1	0	0	0	0	0	0	0	0	0	0	1	1	0
243,411,138	2	0	0	0	0	2	2	1	0	0	1	0	1	0	1	0	0	0	2	1
243,444,450	8	1	4	4	3	0	1	0	0	0	0	0	0	3	1	0	5	1	2	0
251,723,763	3	0	0	0	1	2	1	0	1	0	1	0	1	2	0	0	0	1	2	1
673,710,248	3	0	0	0	3	0	2	0	0	0	0	0	0	1	0	0	0	2	1	0
**686,489,768**	**138**	**35**	**15**	**26**	**44**	**39**	**49**	**10**	**27**	**9**	**28**	**3**	**34**	**51**	**30**	**6**	**45**	**21**	**66**	**29**
707,439,290	2	1	0	1	1	0	1	0	0	0	0	0	0	1	1	0	1	0	1	0
715,696,826	2	0	0	0	1	1	1	0	0	0	0	0	0	0	0	0	0	0	2	0
**715,794,602**	**29**	**4**	**3**	**4**	**15**	**4**	**8**	**4**	**2**	**3**	**3**	**2**	**4**	**8**	**4**	**1**	**7**	**4**	**16**	**5**
715,795,130	4	1	1	1	0	2	2	0	0	0	0	0	0	1	0	0	2	0	2	0
715,827,386	5	1	0	0	2	2	3	0	1	0	1	0	1	1	0	0	1	1	3	1
715,827,890	5	3	1	3	1	0	0	0	0	0	0	0	0	5	0	0	4	0	1	0
**715,827,898**	**112**	**22**	**9**	**14**	**41**	**33**	**43**	**17**	**15**	**12**	**20**	**1**	**31**	**35**	**17**	**3**	**29**	**21**	**59**	**26**
715,827,902	2	0	0	0	1	0	1	0	0	0	0	0	0	0	0	0	0	1	1	0
1,015,188,194	2	0	1	1	1	0	0	0	0	0	0	0	0	1	0	0	1	0	1	0
1,015,196,386	3	2	0	1	1	0	1	0	0	0	0	0	0	0	0	0	2	0	1	0
1,019,412,480	4	0	1	1	2	1	1	0	0	0	0	0	0	2	0	0	1	0	3	0
**1,019,412,672**	**56**	**10**	**3**	**5**	**28**	**14**	**28**	**3**	**10**	**4**	**9**	**0**	**13**	**10**	**8**	**0**	**13**	**15**	**28**	**7**
1,048,619,746	2	1	0	1	0	1	1	1	0	0	1	0	1	0	1	0	1	0	1	0
**1,048,717,506**	**14**	**2**	**1**	**2**	**7**	**3**	**5**	**1**	**3**	**0**	**4**	**0**	**4**	**4**	**4**	**0**	**3**	**1**	**10**	**2**
1,048,718,050	2	1	0	0	1	0	0	0	0	0	0	0	0	0	0	0	1	0	1	0
1,048,742,626	2	0	0	0	1	0	1	0	1	0	1	0	1	1	0	0	0	0	2	1
1,048,750,306	3	1	0	0	2	0	1	0	2	0	2	0	2	0	1	0	1	1	1	1
1,048,750,626	3	0	2	2	0	1	1	0	0	0	0	0	0	3	0	0	2	0	1	0
**1,048,750,818**	**102**	**26**	**8**	**17**	**43**	**22**	**39**	**9**	**22**	**4**	**27**	**1**	**30**	**34**	**16**	**3**	**31**	**23**	**45**	**20**
**1,057,013,475**	**19**	**5**	**5**	**6**	**3**	**6**	**6**	**4**	**2**	**3**	**3**	**0**	**6**	**5**	**7**	**1**	**9**	**3**	**6**	**5**
1,057,029,363	2	0	0	0	0	2	1	0	1	0	1	0	1	0	1	0	0	0	2	0
**1,057,030,131**	**63**	**10**	**6**	**9**	**29**	**12**	**23**	**7**	**11**	**6**	**12**	**0**	**18**	**13**	**13**	**1**	**16**	**11**	**34**	**10**

**Table 2:** Target populations for which molecular-genetic neural nets were trained in order to separate these target populations from their respective counterparts, for example, F2 patients who responded to treatment (in terms of a 40% sustained PANSS-P baseline score reduction) from non-responders

Molecular-genetic Neural Nets: Target Populations				
Subgroup	patients	males	females	mean age [years]
F2 Response	78/29.5%	35	43	33.3±10.0/37.3±9.0
F2 Early improvement	138/52.3%	69	69	34.3±11.2/38.0±9.7
F3 Response	227/35.6%	64	163	44.8±12.2/47.5±11.9
F3 Early improvement	362/57.1%	116	246	44.3±12.6/46.5±13.2
Global side effect score	90/11.7%	28	62	37.3±13.2/40.2±12.2
Sleep disturbances	116/15.0%	43	73	36.8±12.3/43.1±12.2
Cardiovascular disturbances	120/15.6%	54	66	40.5±12.2/43.7±11.6
Weight gain	62/12.7%	33	29	37.9±13.5/43.8±11.5
Elevated IgM levels	75/26.1%	26	49	39.8±13.0/43.7±12.2

About one third of the candidate genes met the criteria for “response genes”.

## Results: response to treatment

Of the initial 1,229 patients enrolled in this study, 902 met the inclusion criteria and had at least three assessments within the first two weeks of treatment (F2 patients: *n* = 264; F3 patients: *n* = 638). Where absolutely necessary, missing assessments were filled in using standard LOCF methods (*last observation carried forward*).

**Fig. 2:**
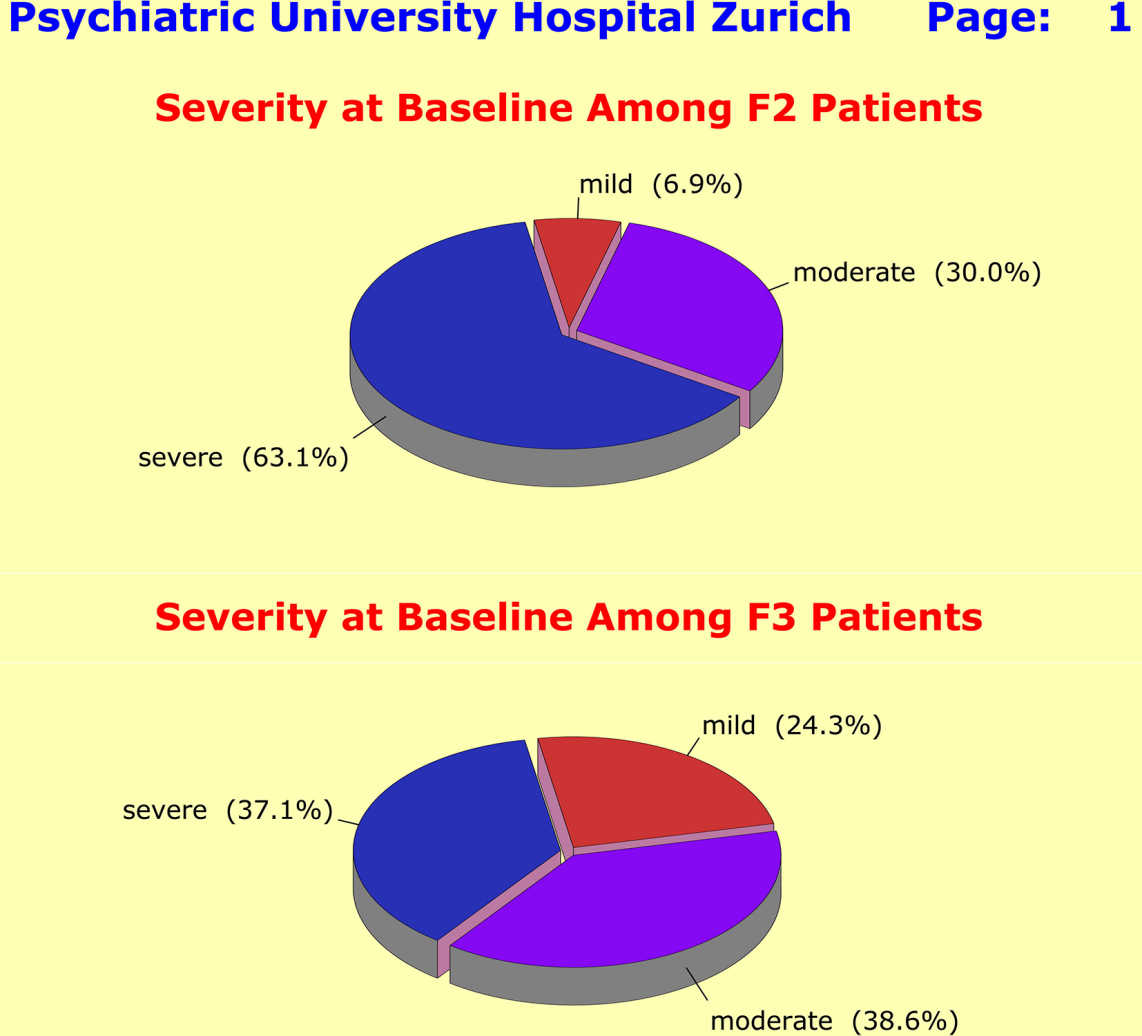
Among the F2 patients there were18 mild cases (PANSS-G score < 30; 6.9%), 79 moderately ill cases (PANSS-G score from 30–40; 30.0%), and 167 severely ill cases (PANSS-G score > 40; 63.1%). By contrast, the rate of severely ill cases was much lower among the F3 patients. There were *n* = 154 mild cases (HAM-D17 score < 20; 24.3%), 248 moderately ill cases (HAM-D17 score from 20–24; 38.6%), and 236 severely ill cases (HAM-D17 score > 24; 63.1%). Over recent years, one has observed a steadily increasing tendency to hospitalize patients with mild depression without true need for such measures

Among the F2 patients, there were 18 mild cases (6.9%), 79 moderately ill cases (30.0%), and 167 severely ill cases (63.1%). By contrast, among the F3 patients the rate of severely ill cases was much lower (*n* = 236; 37.1%), while the rates of mild and moderately ill cases were with *n* = 154 (24.3%) and *n* = 248 (38.6%) significantly higher (Fig. 2).

**Fig. 3:**
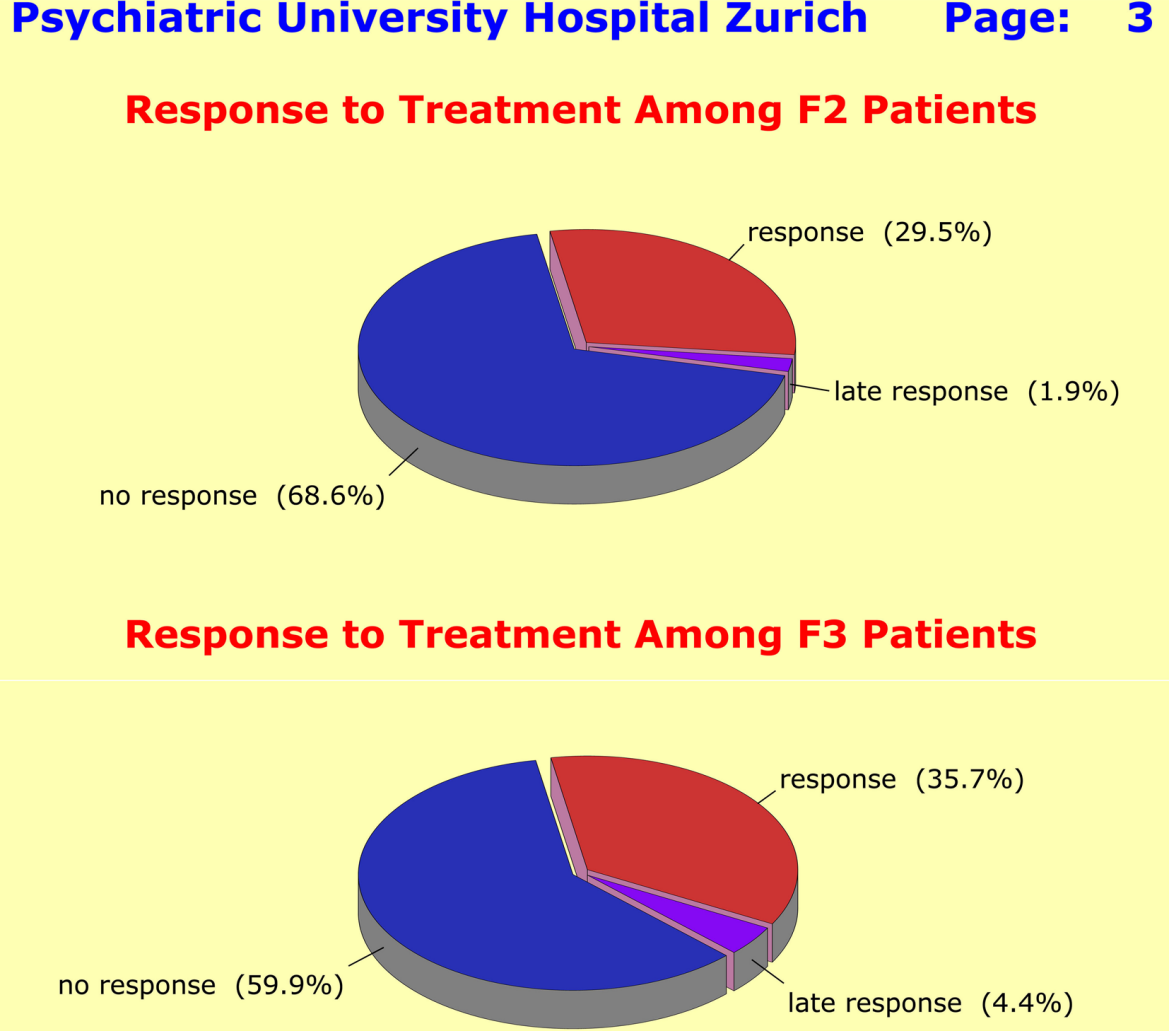
Response to treatment was defined by a sustained 40% PANSS-P baseline score reduction (F2 patients) and by a sustained 50% HAM-D17 baseline score reduction (F3 patients) [Sustained: no deterioration beyond 15% of achieved baseline score reduction]. Clinical data underlined the insufficient effects of current treatment options across F2 and F3 diagnoses. Only a minority of patients met response criteria, a larger proportion got “stuck” after initial improvement, and a considerable number of patients did not respond at all

**Fig. 4:**
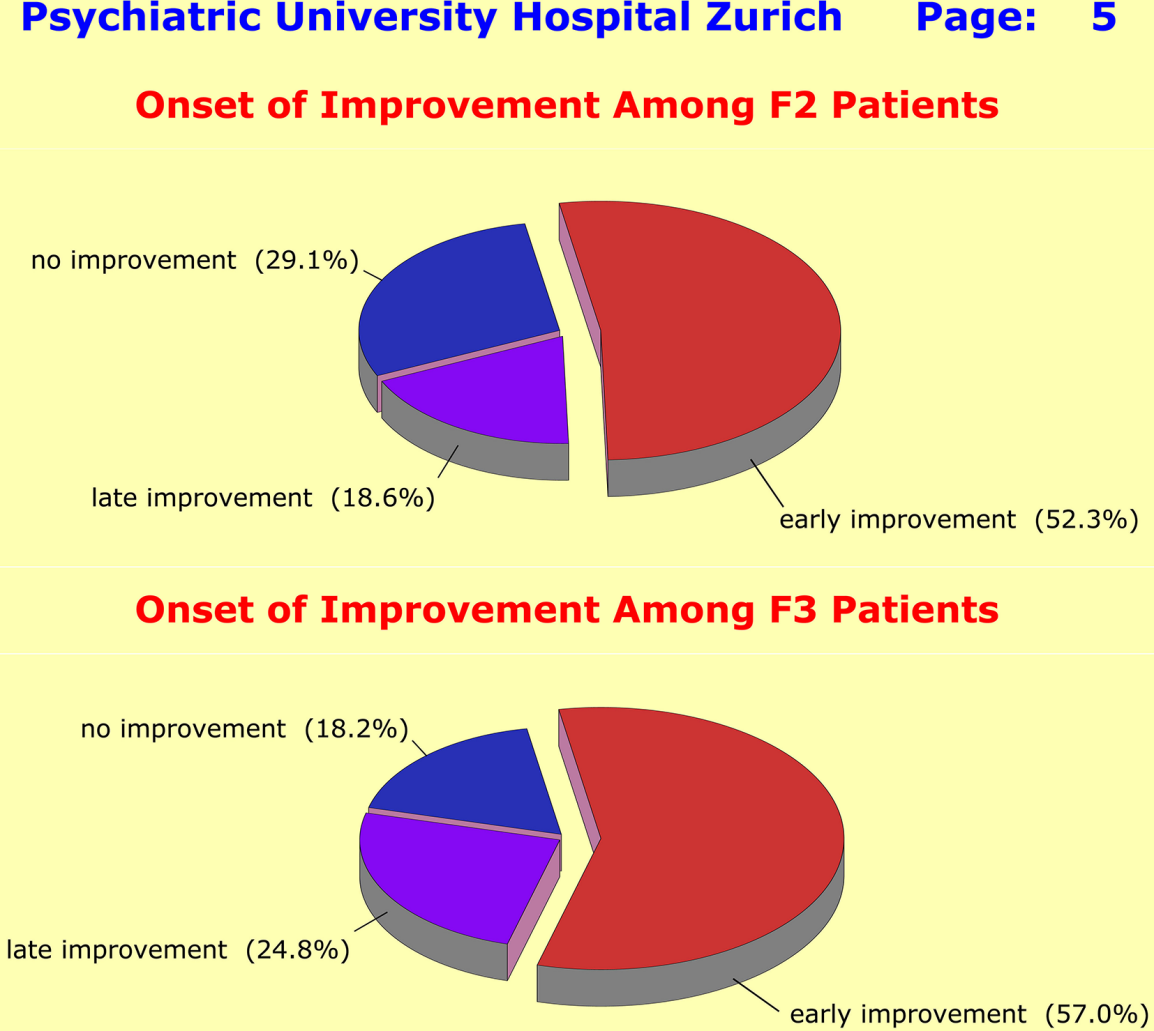
Early improvement was defined by a sustained 20% PANSS-P baseline score reduction (F2 patients) and by a sustained 20% HAM-D17 baseline score reduction (F3 patients) within the first 14 days of treatment [Sustained: no deterioration beyond 15% of achieved baseline score reduction]. The onset of improvement was quite similar across diagnoses, but very heterogeneous across patients. At no point on the 42-day observation timeline was a peak detectable that would indicate a specific onset of action of medication

Despite the significant differences between F2 and F3 patients regarding baseline severity, response characteristics were very similar across the two diagnostic groups: only a minority of patients met the response criteria, a lot of patients got “stuck” after initial improvement, and a considerable number of patients did not improve at all (Fig. 3). The lack of specific treatment effects became particularly evident when one looks at the patients’ time course of improvement: at no point of the 42-day observation period was a peak detectable that would indicate a specific therapeutic effect (Fig. 4).

In the next, tentative step we applied a Generalized Linear Model (GLM) in order to look for significant main effects regarding response to treatment and the development of severe side effects. This analysis, however, did not produce any conclusive results. “No main effect” meant that we could safely exclude the non-informative genes with low between-subject variation from subsequent NNs without concerns for a substantial loss of information.

## Results: neural net analyses

In a first step, we used the multidimensional genotypic patterns of all candidate genes and trained a molecular-genetic Neural Net (NN) that aimed at separating mild from severe cases in terms of baseline severity. This attempt was not successful, neither for the sample as a whole entity, nor in separate analyses of the two diagnostic subsamples.

In the next step, we used the multidimensional genotypic patterns of “response genes” only, and trained a molecular-genetic NN to recognize “treatment responders” under the constraint (“criterion function”) that (1) all “non-responders” were correctly identified^3^; and (2) the number of “false-positive” classified patients in the total sample (across diagnoses) was minimized. Though missing genotype data may not be a problem in single SNP analyses, it can become an unmanageable obstacle in multivariate approaches [17], as is the case with molecular-genetic NNs. We therefore excluded all patients with a missing data rate > 5% from subsequent NN analyses in order to reduce the overall noise level. This precautionary measure resulted in a sample of 771 patients with a missing genotype rate of 2.136±2.7 [%].

In the case of F2 patients, the NN analysis came to a reproducible steady state with 94.7% correctly classified “treatment responders” (71 out of 75; 5.3% false-negatives); 100% correctly classified “non-responders” (*n* = 164); 0.1% false-positives (1 out of 169); and a 29.6% overlap with F3 patients (156 out of 527). In the case of F3 patients, the NN analysis came to a reproducible steady state with 82.6% correctly classified “treatment responders” (157 out of 190; 17.4% false-negatives); 100% correctly classified “non-responders” (*n* = 309); 2.4% false-positives (8 out of 337); and a 35.7% overlap with F2 patients (87 out of 244).

A minimum of 100,000 iterations was required to reach steady states. From 500,000 iterations onwards, the value of the criterion function to be optimized began to oscillate without additional “true” improvements being achieved. The optimum of the criterion function was characterized by a “broad” maximum, made up by several strikingly redundant, mutually interchangeable genes. The redundancy between the genes was due to the fact that disorder-related genotypic patterns were more strongly correlated with each other than one would have expected from the correlations on the gene level. The final F2 and F3 classifiers, constructed under the constraint of reproducibility, were in each case comprised of diagnoses-crossing sets of 5–6 genes out of a pool of 7–12 genes, subsequently referred to as recovery-related “classifier” genes (Tables 3 and 4). There was a major overlap in the range of 80% with the “vulnerability” genes derived by a recent case-control study (*n* = 1,431; [19]).

**Table 3:** Classifier genes have been identified by the NN algorithm as contributing to the separation between F3 responders and non-responders. The NN analysis came to a reproducible steady state with 82.6% correctly classified “treatment responders”, 100% correctly classified “non-responders”, 2.4% false-positives, and a 35.7% overlap with F2 patients. All genetic analyses relied on a genetic-physical map derived from *Ensembl* Build 105 of September 25, 2021

Classifier Genes				
Gene	Chr	Position	SNPs	Gene function
GPR39	2	[132’416’805, 132’646’582]	7	G Protein-coupled receptor 39
STAT4	2	[191’029’576, 191’151’596]	5	Signal transducer and activator of transcription 4
SLC6A1	3	[10’992’186, 11’039’247]	6	Solute carrier family 6 member 1
GRIA1	5	[153’489’615, 153’813’869]	7	Glutamate ionotropic receptor AMPA type subunit 1
GABRR1	6	[ 89’177’504, 89’231’278]	6	Gamma-aminobutyric acid type A receptor subunit rho 1
GRIA4	11	[105’609’994, 105’982’092]	6	Glutamate ionotropic receptor AMPA type subunit 4
NCAM	11	[113’265’137, 113’274’032]	6	Neural cell adhesion molecule

**Table 4:** Classifier genes have been identified by the NN algorithm as contributing to the separation between F2 responders and non-responders. The NN analysis came to a reproducible steady state with 94.7% correctly classified “treatment responders”, 100% correctly classified “non-responders”, 0.1% false-positives, and a 29.6% overlap with F3 patients. All genetic analyses relied on a genetic-physical map derived from *Ensembl* Build 105 of September 25, 2021

Classifier Genes				
Gene	Chr	Position	SNPs	Gene function
GRIK3	1	[36’795’527, 37’034’515]	6	Glutamate ionotropic receptor kainate type subunit 3
POMC	2	[25’160’853, 25’168’903]	4	Proopiomelanocortin
GPR39	2	[132’416’805, 132’646’582]	7	G Protein-coupled receptor 39
STAT1	2	[190’908’460, 191’020’960]	6	Signal transducer and activator of transcription 1
STAT4	2	[191’029’576, 191’151’596]	5	Signal transducer and activator of transcription 4
SLC6A1	3	[10’992’186, 11’039’247]	6	Solute carrier family 6 member 1
SLC6A3	5	[1’392’794, 1’445’440]	5	Solute carrier family 6 member 3
GRIA1	5	[153’489’615, 153’813’869]	7	Glutamate ionotropic receptor AMPA type subunit 1
NRG1	8	[31’639’222, 32’855’666]	5	Neuregulin 1
BDNF	11	[27’654’893, 27’722’058]	5	Brain derived neurotrophic factor
GRIK4	11	[120’511’746, 120’988’906]	5	Glutamate ionotropic receptor kainate type subunit 4
HTR2A	13	[46’831’546, 46’897’076]	4	5-hydroxytryptamine receptor 2 A

Contrary to expectations, the reverse methodical approach did not succeed, namely to train a molecular-genetic NN to recognize “treatment non-responders” under the constraint that all “treatment responders” were correctly identified, while the number of “false-positive” classified patients in the total sample (across diagnoses) was minimized. Though the iterative optimization of the criterion function reached steady states and suggested an acceptable classifier performance, the resulting diagnostic overlap was unacceptably high in the range of 60% or more. Apparently, it was not possible to “learn” the molecular-genetic characteristics of “treatment-responders” from those of “non-responders”.

Finally, we trained molecular-genetic NNs to recognize “early improvers” as target population under the constraint that all “late improvers” and “non-improvers” were correctly identified, while the number of “false-positive” classified patients in the total sample was minimized. It turned out quite quickly that the envisaged molecular-genetic NNs did not come to a useful steady state solution as long as the control group was comprised of the combination of “late improvers” and “non-improvers”. Therefore, we excluded all “late improvers” from subsequent analyses in order to sharpen the contrast between target- and control population. Despite these precautionary measures, we could not find a molecular-genetic NN for the F2 patients that reliably separated “early improvers” from “non-improvers” (and “late improvers”). A possible reason for this could be that the PANSS-P criterion did not adequately measure the “true” onset of improvement as it ignores general psychopathology and negative symptoms.

By contrast, in the case of F3 patients the NN analysis using the HAM-D17 criterion came to a reproducible steady state with 88.0% correctly classified “non-improvers” (81 out of 92; 12.0% false-negatives); 100% correctly classified “early improvers” (*n* = 289); 12.2% false-positives (29 out of 238); and a 16.0% overlap with F2 patients (39 out of 244). The final classifier, constructed under the constraint of reproducibility, was comprised of a set of five genes out of a pool of 7 genes (Table 5). Again, the classifier genes overlapped to a larger extent with those related to recovery and vulnerability.

**Table 5:** Classifier genes have been identified by the NN algorithm as contributing to the separation between F3 early responders (within first 2 weeks) and non-responders. The NN analysis came to a reproducible steady state with 88.0% correctly classified “no improvers”, 100% correctly classified “early improvers”, 12.2% false-positives, and a 16.0% overlap with F2 patients. The attempt to separate F2 early responders from “no-improvers” by NN analysis failed. All genetic analyses relied on a genetic-physical map derived from *Ensembl* Build 105 of September 25, 2021

Classifier Genes				
Gene	Chr	Position	SNPs	Gene function
PRDM2	1	[13’700’188, 13’825’079]	6	PR domain zinc finger protein 2
GPR39	2	[132’416’805, 132’646’582]	7	G Protein-coupled receptor 39
SLC6A1	3	[10’992’186, 11’039’247]	6	Solute carrier family 6 member 1
GRID2	4	[92’303’966, 93’810’157]	5	Glutamate ionotropic receptor delta type subunit 2
GRIA1	5	[153’489’615, 153’813’869]	7	Glutamate ionotropic receptor AMPA type subunit 1
ABCB1	7	[87’503’017, 87’713’323]	8	ATP binding cassette subfamily B member 1
ADAM22	7	[87’934’143, 88’202’889]	8	ADAM metallopeptidase domain 22

The reverse methodical approach did not succeed, namely to train a molecular-genetic NN to recognize “non-improvers” under the constraint that all “early improvers” were correctly identified, while the number of “false-positive” classified patients in the total sample (across diagnoses) was minimized. Apparently, it was not possible to “learn” the molecular-genetic characteristics of “non-improvers” from those of “early improvers”.

## Results: random splitting

The F3 sample of more than 600 patients was large enough to allow for random splitting tests (repeated split-half technique). Random-splitting repeated 20 times, —under the constraint of reproducibility within and across half-splits—, led to a general drop of correctly classified patients in the range of 10%. On the other hand, no subsamples were detected for which the proposed method of approach entirely failed.

## Results: elevated IgM levels

In this step, we trained molecular-genetic NNs to recognize “patients with elevated IgM levels” under the constraint that all “patients with normal IgM levels” were correctly identified, while the number of “false-positive” classified patients in the total sample was minimized. Using various IgM thresholds, the optimization came to steady states of 0% false-negative and some 25-28% false-positive classifications. The final classifier, constructed under the constraint of reproducibility, was comprised of a set of 5–6 genes out of a pool of 8 genes (Table 6).

**Table 6:** Classifier genes have been identified by the NN algorithm as contributing to the separation between patients with elevated IgM levels and patients with normal values. The NN analysis came to a reproducible steady state with 100% correctly classified “elevated IgM levels”, 72% 75% correctly classified “normal values”, and 25% 28% false-positives. All genetic analyses relied on a genetic-physical map derived from *Ensembl* Build 105 of September 25, 2021

Classifier Genes				
Gene	Chr	Position	SNPs	Gene function
GPR39	2	[132’416’805, 132’646’582]	7	G Protein-coupled receptor 39
SLC6A1	3	[ 10’992’186, 11’039’247]	6	Solute carrier family 6 member 1
GRIA1	5	[153’489’615, 153’813’869]	7	Glutamate ionotropic receptor AMPA type subunit 1
ABCB1	7	[ 87’503’017, 87’713’323]	8	ATP binding cassette subfamily B member 1
ADAM22	7	[ 87’934’143, 88’202’889]	8	ADAM metallopeptidase domain 22
NRG1	8	[ 31’639’222, 32’855’666]	5	Neuregulin 1
NCAM	11	[113’265’137, 113’274’032]	6	Neural cell adhesion molecule
ADRA1D	20	[4’220’630, 4’249’287]	5	Adrenoceptor alpha 1D

## Results: drug-induced weight gain

Our tentative analyses regarding the genetic predisposition to overweight and to unwanted weight gain under psychopharmacological treatment did not lead to clear and conclusive results. One possible reason for this negative outcome could be that the genes with major contributions to overweight and unwanted weight gain were actually not included in the analysis. We will therefore address this topic in a more comprehensive way as part of our upcoming molecular-genetic study on the side-effect clusters «sleep», «appetite», «sexuality», «gastro-intestinal», «cardiac-respiratory», «autonomic», «psychosomatic», «neurological», and «cardiovascular» disturbances under psychopharmacological treatment.

## Results: biological ethnicity

In a tentative analysis, we evaluated the patients’ “biological ethnicity”. While GWASs are focusing on global, genome-wide “genetic ancestry” to estimate the probability of a subject to belong to a certain “ancestry group” [48], the concept of “biological ethnicity” has its focus on hidden population stratifications that arise locally within chromosome segments [19].

We chose the CLOCK gene for analyzing “biological ethnicity” because this gene likely shows distinctive adaptations to typical “North-South” and “West-East” diurnal and seasonal patterns that might give rise to population stratification. We created five gene vectors by subdividing the gene into five segments, each with 15 SNPs, and applied cluster analyses for the detection of “natural” subgroups inherent in the patients’ gene vectors.

A principal component analysis prior to cluster analysis eliminated the correlations between the five CLOCK gene segments almost completely. The first two eigenvalues already explained 96.9% of the observed variance, so that subsequent cluster analyses were carried out solely with the two corresponding eigenvectors. We found 3 clearly separated clusters, but these were unrelated to the patients’ response characteristics and clinical diagnoses.

## Discussion

Based on a sample of quite respectable size we addressed the question of predicting response to psychopharmacological treatment by means of a newly developed method of approach. Unlike conventional association methods where millions of statistical tests have to be carried out to detect a few tiny signals, and where the term “underpowered study” often has to serve as explanation for not detecting reliable signals, the multivariate approach of this study works completely different. Multidimensional “gene vectors”, assembled from the genotypes of 4–8 polymorphic SNPs located within genes, assess genetic diversity through the genes’ highly distinctive “fingerprints” that exhibit plentiful between-subject variation. In consequence, “gene vectors” have a much higher resolution than single SNP approaches when it comes to detailing subtle between-subject differences. Therefore, the difficulties caused by “underpowered” studies hardly ever become relevant: small sample sizes are in many cases sufficient to address clinical questions. Most notably, “gene vectors” can reveal complex structures of SNP data that are not detectable by conventional association methods. However, this comes at the cost of (1) an elevated risk of overfitting in machine-learning models; and (2) an increased possibility of loss of generalizability if hidden sampling biases go undetected.

NN overfitting can be largely avoided by *k*-fold cross-validation that splits the data into *k* roughly equal parts, using *k*-1 partitions for training, while one partition is used for testing. This process is repeated until each partition has served as a testing set, so that *k* estimates of prediction errors are generated. The resulting prediction errors are approximately unbiased for the “true” error for sufficiently large *k*. Furthermore, the well-proven “random walk” strategy was used in order to distinguish between local and global minima. With *k* = 10, NN overfitting was effectively ruled out in this study, that is, the model was iteratively adjusted so that all “new” cases of the 10 test-subsamples (each comprising 10% of the total sample) were classified as correctly as possible. Thus, results were de facto replicated in 10 completely independent samples.

The inpatients of this study were recruited from the daily admissions at five residential mental health treatment centers in Switzerland and Southern Germany (F2 patients: *n* = 264; F3 patients: *n* = 638). Accordingly, we can be quite confident that our patient sample is fairly representative for Central Europe so that the classifiers derived through this study can be expected to handle newly added cases from Central Europe with only slightly reduced performance. On the other hand, it is obvious that the addition of cases from Asia, South America or Africa will likely involve previously unknown “response genes” as well as previously unknown genotypic patterns of already known “response genes”, thus making the re-training of the NN models unavoidable.

Machine learning models are very prone to picking up genetic ancestry effects in case-control studies. This is particularly true for GWASs based on the aggregation of various samples, where hidden population stratification due to admixture of people with different ancestries can give rise to grossly misleading signals [48]. The situation is completely different when it comes to therapy response. Clinical response data from thousands of patients of European descent show no influence of ancestry effects at all [38–40]. If there were a “true” link between ancestry and therapy response, this would certainly have been revealed a long time ago.

The discovery of “response genes” and their use as a-priori knowledge in the NN analyses ultimately enabled the successful construction of classifiers that reproducibly separated responders from non-responders, using *k*-fold cross-validation. If all “response genes” were excluded, NNs based on the remaining genes did not lead to reliable prediction models with acceptable classification rates. Therefore, “response genes” represent a key result of this study and underline the role of the patients’ «recovery disposition» regarding therapy response. In particular, “response genes” enabled classifiers to reproducibly separate non-responders from responders, whereas NNs based on gene sets in the absence of “response genes” did not lead to reliable prediction models: Useful classifiers in the field of therapy response did not seem to be constructible from ARBITRARY sets of genes. Although additional “response genes” undoubtedly exist, identifying those genes is not that simple, because the respective search is becoming more and more cumbersome the more “response genes” have already been found. Moreover, the most promising candidates had already been selected in preparation for this study.

This study did not aim at constituting a polygenic model that “explains” treatment response in a direct and causal way. Rather, it was assumed that patients possess an unspecific physical-genetic disposition that enables, facilitates, impedes or prevents recovery from major psychiatric disorders by setting various thresholds for exogenous triggers that can initiate improvement. Our results convincingly support the existence of such a «recovery disposition». This in line with the clinical data of thousands of patients under monotherapy, suggesting that psychopharmacological treatments are all non-causal and appear to act unspecifically by triggering the onset of recovery in a subgroup of patients, irrespective of their biochemical design and primary site of pharmacological action. Once triggered, the recovery of patients follows its “natural” course as equally observed under placebo treatment [37–40].

The disadvantage of genetic diversity models, however, is that they do not allow any direct conclusions to be drawn about the causes of psychiatric disorders. The resulting classifiers can nonetheless be used in the sense of a “surrogate”, since clinicians are also highly interested in reliable tools that can “do the job” even if etiology and pathogenesis of the treated disorders remain unknown. The observed overlap between clinical diagnoses on the genotype level gets support by a number of observations on the phenotype level: (1) no homotypic diagnostic patterns are observed in families with multiple affected subjects; (2) there appears to be a continuum between affective and psychotic disorders [49, 50]; (3) a majority of patients with schizophrenia diagnoses also reports major depressive symptoms; and (4) the diagnoses of monozygotic twins who both suffer from psychiatric disorders can be quite different even though they share the same genome [51].

The classification of patients in terms of IgM levels, though successful, was somewhat inconclusive overall, as the false-positive classification errors always lay in the range of 25–28%, irrespective of the chosen IgM threshold. This suggested that IgM level alone may be an insufficient measure when assessing aberrancies of the inflammatory response system. It is an open question here whether elevated IgM levels are produced by the immune system in response to a perceived threat, or are the result of an aberrant immune response of the organism against its own cells and tissues (autoimmune inflammatory processes) [41].

It was not really surprising that no molecular-genetic NNs could be fitted to the clinical quantity “severity at baseline”, mainly because this quantity is somewhat vague and can fluctuate considerably within patients across recurrent episodes. The negative outcome regarding the clinical quantity “onset of improvement” among the F2 patients could also be expected, since this quantity is insufficiently captured by PANSS-P scores that do not take general psychopathology and negative symptoms into account.

Response to treatment was quite similar across diagnoses but very heterogeneous across patients. At no point on the 42-day observation period was a peak detectable that would indicate a specific medication effect. Furthermore, there were no major differences between the various treatment modalities except for the fact that response rates were significantly lower under polypharmacy in comparison to monotherapy.

This project is to be regarded as a pilot study and needs independent replication. Given the robustness of our results, we can expect that new and independent studies will yield similar results for patients from Central European. By contrast, the analysis of patients from Asia, South America, or Africa will likely involve previously unknown “response genes” as well as previously unknown genotypic patterns of already known “response genes”. At first glance, existing GWASs with large samples of patients appear to be a good basis for replicating and extending our results. However, GWASs typically have relatively high error rates along with high percentages of missing data. This may not be a major problem in single SNP analyses, but can become an unmanageable obstacle in multivariate approaches [17]. Another problem arises from the fact that the SNPs of GWASs are fixed and cannot be freely chosen within genes as needed. In consequence, the idea of using GWAS data as an abundant and straight-forward resource for identifying response genes and analyzing the genes’ role in treatment response does not appear to be particularly promising.

## Conclusions

The model presented here for predicting the response to psychopharmacological treatment has been convincingly confirmed by empirical data. The respective results are pretty robust so that the model should be easily verified through independent studies. Even though the proposed method of approach did not follow mainstream ideas, it nevertheless deserves attention. In particular, since the observed overlap between (1) recovery-related genes (“recovery disposition”), (2) vulnerability-related genes (“resilience”), and (3) inflammation-related genes, is likely to provide new insights into the etiology and pathogenesis of major psychiatric disorders, thus leading to better treatments to the benefit of patients.

### Limitations

The majority of patients came from Central Europe, so that the variation in biological ethnicity was modest. The molecular-genetic NN classifiers derived through this sample may not necessarily show the same good performance with ethnically different populations, as the weight matrices involved appear to be population-dependent.

## Electronic supplementary material

Below is the link to the electronic supplementary material.

Supplementary Material 1

Supplementary Material 2

## Appendix

### Schizophrenia GWASs

The most sophisticated approach to explaining associations detected by GWASs is by the “Schizophrenia Working Group” of the Psychiatric Genetics Consortium [13]. This approach relies on a combination of fine-mapping, transcriptomic analysis, and functional genomic annotations. The authors reported 120 prioritized loci distributed over the entire genome (with the exception of chromosome 22): 88 intron-, 16 intergenic-, 4 missense-, 3 regulatory region-, 2 splice donor-, two 3 prime UTR-, two 5 prime UTR-, 1 non-coding transcript exon-, and 2 synonymous variants. The clinical significance of these results is still unclear, and practical applications do not appear to be within reach.

### Major depression GWASs

In their meta-analysis of seven cohorts, the “Major Depressive Disorder Working Group” of the Psychiatric Genomics Consortium [12] reported 44 prioritized loci distributed over 18 chromosomes: 27 intron-, 12 intergenic-, 2 regulatory region-, one 3 prime UTR-, and 2 non-coding transcript exon variants. The clinical significance of these results is still unclear, and practical applications do not appear to be within reach.

### Bipolar disorder GWASs

A review of 15 GWASs yielded a list of 67 loci distributed over 18 chromosomes [14]: 43 intron-, 12 intergenic-, three 3 prime UTR-, two 5 prime UTR-, 3 regulatory region-, 1 splice acceptor-, 1 synonymous-, and 2 non-coding transcript exon variants. The clinical significance of these results is still unclear, and practical applications do not appear to be within reach.

### Alzheimer’s disease GWASs

In a review article of 3 recent GWASs in comparison to the meta-analysis carried out by the International Genomics of Alzheimer’s Project (IGAP), the authors reported 77 loci distributed over 18 chromosomes [15]: 41 intron-, 14 intergenic-, 8 regulatory region-, three 3 prime UTR-, 5 missense-, 3 TF binding site-, 1 stop gained-, and 2 non-coding transcript exon variants. The newer GWAS were not independent of each other, yet produced some inconsistent outcomes. The clinical significance of these results is still unclear, and practical applications do not appear to be within reach.

### Antipsychotic response GWASs

In a review article of GWAS studies that used quantitative measures of antipsychotic response [11], the authors found that of 20 genome-wide significant SNPs identified by these studies, only two (rs11722719 near PCDH7 and rs1875705 in GRID2), replicated across independent datasets. And they concluded “while perhaps the most parsimonious conclusion that can be drawn from these findings is largely a failure in replication, there is some room for optimism as the findings continue to support a role for glutamate system genes in treatment response to drugs with primary pharmacodynamic effects on D2 receptors”.

## Footnotes

1 It is based on the model that mental health is the result of a complex self-regulating dynamic system with many degrees of freedom. The system reacts adequately to endogenous and exogenous challenges and handles these successfully. Psychiatric disorders can develop when parts of this dynamic system do no longer react adequately to the many challenges to which it is constantly exposed. Which parts fail can vary from patient to patient, that is, similar clinical pictures can have different causes. Recovery means bringing the dynamic system fully back into function and self-regulation.

2 sustained: no deterioration beyond 15% of achieved baseline score reduction.

3 This is because “non-responders” are the patients who can be identified more reliably than “responders”.
