# Supplementary material for: Genetic determinants of antidepressant and antipsychotic drug response
Source: Eur Arch Psychiatry Clin Neurosci. 2024 Oct 9;275(5):1419–35. doi: 10.1007/s00406-024-01918-5 (PMC12271245; doi:10.1007/s00406-024-01918-5)
Supplement: Supplementary file 1 — Supplementary Material 1 [file 406_2024_1918_MOESM1_ESM.docx]

**Supplementary Table 1**: List of candidate genes analyzed within the scope of this project, continued in Supplementary Table 2. The list of SNPs and the classifiers as SAS 9.4 macros are available on request.

| **List of Genes (Part I)** | | | |
| --- | --- | --- | --- |
| **Gene** | **Chr** | **Position Ensembl Build 105, 9/25/2021** | **Function** |
| **PRDM2** | 1 | [ 13'700'188, 13'825'079] | PR domain zinc finger protein 2 |
| **OPRD1** | 1 | [ 28'812'170, 28'871'267] | Delta-type opioid receptor |
| **GRIK3** | 1 | [ 36'795'527, 37'034'515] | Glutamate ionotropic receptor kainate type subunit 3 |
| **CYP4B1** | 1 | [ 46'757'838, 46'819'413] | Cytochrome P450 family 4 subfamily B member 1 |
| **CYP2J2** | 1 | [ 59'893'308, 59'926'773] | Cytochrome P450 family 2 subfamily J member 2 |
| **ADAR** | 1 | [154'581'695, 154'628'013] | Adenosine deaminase RNA specific |
| **APOB** | 2 | [ 21'001'429, 21'044'073] | Apolipoprotein B |
| **POMC** | 2 | [ 25'160'853, 25'168'903] | Proopiomelanocortin |
| **CYP1B1** | 2 | [ 38'066'973, 38'109'902] | Cytochrome P450 family 1 subfamily B member 1 |
| **GPR39** | 2 | [132'416'805, 132'646'582] | G Protein-coupled receptor 39 |
| **LYPD1** | 2 | [132'643'286, 132'671'579] | LY6/PLAUR domain containing protein 1 |
| **GAD1** | 2 | [170'813'213, 170'861'151] | Glutamate decarboxylase 1 |
| **STAT1** | 2 | [190'908'460, 191'020'960] | Signal transducer and activator of transcription 1 |
| **STAT4** | 2 | [191'029'576, 191'151'596] | Signal transducer and activator of transcription 4 |
| **CYP27A1** | 2 | [218'781'749, 218'815'293] | Cytochrome P450 family 27 subfamily A member 1 |
| **SLC4A3** | 2 | [219'627'394, 219'641'980] | Solute carrier family 4 member 3 |
| **SLC6A1** | 3 | [ 10'992'186, 11'039'247] | Solute carrier family 6 member 1 |
| **SLC6A6** | 3 | [ 14'402'576, 14'489'349] | Solute carrier family 6 member 6 |
| **CHMP2B** | 3 | [ 87'227'271, 87'255'556] | Charged multivesicular body protein 2b |
| **DRD3** | 3 | [114'127'580, 114'199'407] | Dopamine receptor D3 |
| **GABRA2** | 4 | [ 46'243'548, 46'475'230] | Gamma-aminobutyric acid receptor subunit alpha 2 |
| **GABRA4** | 4 | [ 46'918'900, 46'993'581] | Gamma-aminobutyric acid receptor subunit alpha 4 |
| **GABRB1** | 4 | [ 46'993'723, 47'426'447] | Gamma-aminobutyric acid receptor subunit beta 1 |
| **CLOCK_a** | 4 | [ 55'427'903, 55'546'909] | Clock circadian regulator |
| **CLOCK_b** | 4 | [ 55'427'903, 55'546'909] | Clock circadian regulator |
| **CLOCK_c** | 4 | [ 55'427'903, 55'546'909] | Clock circadian regulator |
| **CLOCK_d** | 4 | [ 55'427'903, 55'546'909] | Clock circadian regulator |
| **CLOCK_e** | 4 | [ 55'427'903, 55'546'909] | Clock circadian regulator |
| **GRID2** | 4 | [ 92'303'966, 93'810'157] | Glutamate ionotropic receptor delta type subunit 2 |
| **PGRMC2** | 4 | [128'269'237, 128'288'829] | Membrane-associated progesterone receptor component 2 |
| **MTNR1A** | 4 | [186'533'655, 186'555'567] | Melatonin receptor type 1A |
| **SLC6A3** | 5 | [ 1'392'794, 1'445'440] | Solute carrier family 6 member 3 |
| **HTR1A** | 5 | [ 63'957'874, 63'962'507] | 5-Hydroxytryptamine receptor 1A |
| **GRIA1** | 5 | [153'489'615, 153'813'869] | Glutamate ionotropic receptor AMPA type subunit 1 |
| **ADRA1B** | 5 | [159'865'080, 159'973'012] | Adrenoceptor alpha 1B |
| **GABRA1** | 5 | [161'847'063, 161'899'981] | Gamma-aminobutyric acid type A receptor subunit alpha 1 |
| **GABRG2** | 5 | [162'000'057, 162'162'977] | Gamma-aminobutyric acid type A receptor subunit gamma 2 |
| **TNF** | 6 | [ 31'575'565, 31'578'336] | Tumor necrosis factor |
| **NEU1** | 6 | [ 31'857'659, 31'862'905] | Neuraminidase 1 |
| **NOTCH4** | 6 | [ 32'194'843, 32'224'067] | Notch receptor 4 |
| **HLADRB1** | 6 | [ 32'459'821, 32'473'500] | Major histocompatibility complex, class II, DR beta 1 |
| **GRM4** | 6 | [ 34'018'643, 34'155'622] | Glutamate metabotropic receptor 4 |
| **HTR1B** | 6 | [ 77'460'924, 77'463'491] | 5-hydroxytryptamine receptor 1B |
| **GABRR1** | 6 | [ 89'177'504, 89'231'278] | Gamma-aminobutyric acid type A receptor subunit rho 1 |
| **GABRR2** | 6 | [ 89'254'464, 89'315'299] | Gamma-aminobutyric acid type A receptor subunit rho 2 |
| **MCHR2** | 6 | [ 99'918'519, 99'994'247] | Melanin concentrating hormone receptor 2 |
| **GRIK2** | 6 | [100'962'701, 102'081'622] | Glutamate ionotropic receptor kainate type subunit 2 |
| **GRM1** | 6 | [146'027'646, 146'437'601] | Glutamate metabotropic receptor 1 |
| **TBP** | 6 | [170'554'302, 170'572'870] | TATA-box binding protein |
| **CRHR2** | 7 | [ 30'651'942, 30'700'129] | Corticotropin releasing hormone receptor 2 |
