# Supplementary material for: Genetic determinants of antidepressant and antipsychotic drug response
Source: Eur Arch Psychiatry Clin Neurosci. 2024 Oct 9;275(5):1419–35. doi: 10.1007/s00406-024-01918-5 (PMC12271245; doi:10.1007/s00406-024-01918-5)
Supplement: Supplementary file 2 — Supplementary Material 2 [file 406_2024_1918_MOESM2_ESM.docx]

**Supplementary Table 2**: List of candidate genes analyzed within the scope of this project, continuation of Supplementary Table 1.

| **List of Genes (Part II)** | | | |
| --- | --- | --- | --- |
| **Gene** | **Chr** | **Position Ensembl Build 105, 9/25/2021** | **Function** |
| **ABCB1** | 7 | [ 87'503'017, 87'713'323] | ATP binding cassette subfamily B member 1 |
| **ADAM22** | 7 | [ 87'934'143, 88'202'889] | ADAM metallopeptidase domain 22 |
| **CYP3A5** | 7 | [ 99'648'194, 99'679'998] | Cytochrome P450 family 3 subfamily A member 5 |
| **CYP3A7** | 7 | [ 99'705'036, 99'735'196] | Cytochrome P450 family 3 subfamily A member 7 |
| **CYP3A4** | 7 | [ 99'756'960, 99'784'248] | Cytochrome P450 family 3 subfamily A member 4 |
| **LRRN3** | 7 | [111'091'006, 111'125'454] | Leucine rich repeat neuronal 3 |
| **ADRA1A** | 8 | [ 26'748'150, 26'867'278] | Adrenoceptor alpha 1A |
| **NRG1** | 8 | [ 31'639'222, 32'855'666] | Neuregulin 1 |
| **CRH** | 8 | [ 66'176'376, 66'178'464] | Corticotropin releasing hormone |
| **DBH** | 9 | [133'636'363, 133'659'329] | Dopamine beta-hydroxylase |
| **CYP2C18** | 10 | [ 94'683'729, 94'736'190] | Cytochrome P450 family 2 subfamily C member 18 |
| **CYP2C19** | 10 | [ 94'762'681, 94'855'547] | Cytochrome P450 family 2 subfamily C member 19 |
| **CYP2C9** | 10 | [ 94'938'658, 94'990'091] | Cytochrome P450 family 2 subfamily C member 9 |
| **TH** | 11 | [ 2'163'929, 2'171'815] | Tyrosine hydroxylase |
| **TPH1** | 11 | [ 18'017'555, 18'046'269] | Tryptophan hydroxylase 1 |
| **SLC6A5** | 11 | [ 20'599'594, 20'659'285] | Solute carrier family 6 member 5 |
| **BDNF** | 11 | [ 27'654'893, 27'722'058] | Brain derived neurotrophic factor |
| **MTNR1B** | 11 | [ 92'969'651, 92'985'066] | Melatonin receptor 1B |
| **GRIA4** | 11 | [105'609'994, 105'982'092] | Glutamate ionotropic receptor AMPA type subunit 4 |
| **NCAM** | 11 | [113'265'137, 113'274'032] | Neural cell adhesion molecule |
| **DRD2** | 11 | [113'409'605, 113'475'691] | Dopamine receptor D2 |
| **HTR3B** | 11 | [113'904'796, 113'949'079] | 5-hydroxytryptamine receptor 3B |
| **HTR3A** | 11 | [113'975'075, 113'990'313] | 5-hydroxytryptamine receptor 3A |
| **GRIK4** | 11 | [120'511'746, 120'988'906] | Glutamate ionotropic receptor kainate type subunit 4 |
| **GRIN2B** | 12 | [ 13'437'942, 13'982'002] | Glutamate ionotropic receptor NMDA type subunit 2B |
| **HTR2A** | 13 | [ 46'831'546, 46'897'076] | 5-hydroxytryptamine receptor 2A |
| **GABRB3** | 15 | [ 26'543'546, 26'939'539] | Gamma-aminobutyric acid type A receptor subunit beta 3 |
| **GABRA5** | 15 | [ 26'866'911, 26'949'208] | Gamma-aminobutyric acid type A receptor subunit alpha 5 |
| **CHRNA7** | 15 | [ 31'923'438, 32'173'018] | Cholinergic receptor nicotinic alpha 7 subunit |
| **SRP14** | 15 | [ 40'035'690, 40'039'181] | Signal recognition particle 14 |
| **CYP1A1** | 15 | [ 74'719'542, 74'725'536] | Cytochrome P450 family 1 subfamily A member 1 |
| **CYP1A2** | 15 | [ 74'748'845, 74'756'607] | Cytochrome P450 family 1 subfamily A member 2 |
| **AKAP13** | 15 | [ 85'380'571, 85'749'358] | A-kinase anchoring protein 13 |
| **SLC6A2** | 16 | [ 55'655'988, 55'707'645] | Solute carrier family 6 member 2 |
| **C16orf3** | 16 | [ 90'028'908, 90'029'901] | GAS8 antisense RNA 1 |
| **SLC6A4** | 17 | [ 30'194'319, 30'236'002] | Solute carrier family 6 member 4 |
| **CACNA1A** | 19 | [ 13'206'442, 13'633'025] | Calcium voltage-gated channel subunit alpha 1A |
| **CYP2B6** | 19 | [ 40'991'282, 41'018'398] | Cytochrome P450 family 2 subfamily B member 6 |
| **APOE** | 19 | [ 44'905'791, 44'909'393] | Apolipoprotein E |
| **NLRP7** | 19 | [ 54'923'509, 54'966'312] | NLR family pyrin domain containing 7 |
| **ADRA1D** | 20 | [ 4'220'630, 4'249'287] | Adrenoceptor alpha 1D |
| **GRIK1** | 21 | [ 29'536'933, 29'940'033] | Glutamate ionotropic receptor kainate type subunit 1 |
| **COMT** | 22 | [ 19'941'371, 19'969'975] | Catechol-O-methyltransferase |
| **NF2** | 22 | [ 29'603'556, 29'698'598] | Moesin-ezrin-radixin like (MERLIN) tumor suppressor |
| **MCHR1** | 22 | [ 40'679'273, 40'682'812] | Melanin concentrating hormone receptor 1 |
| **CYP2D6** | 22 | [ 42'126'499, 42'130'865] | Cytochrome P450 family 2 subfamily D member 6 |
| **MAOA** | 23 | [ 43'654'907, 43'746'817] | Monoamine oxidase A |
| **MAOB** | 23 | [ 43'766'610, 43'882'450] | Monoamine oxidase B |
| **HTR2C** | 23 | [114'584'078, 114'910'061] | 5-hydroxytryptamine receptor 2C |
| **GRIA3** | 23 | [123'184'153, 123'490'915] | Glutamate ionotropic receptor AMPA type subunit 3 |
